# A New Insight Into p53-Inhibiting Genes in Epstein–Barr Virus-Associated Gastric Adenocarcinoma

**DOI:** 10.52547/ibj.3784

**Published:** 2022-08-30

**Authors:** Seyed Mohammad Ali Hashemi, Abdolvahab Moradi, Seyed Younes Hosseini, Hadi Razavi Nikoo, Taravat Bamdad, Zahra Faghih, Jamal Sarvari, Alijan Tabarraei

**Affiliations:** 1 Department of Microbiology, School of Medicine, Golestan University of Medical Sciences, Gorgan, Iran;; 2 Department of Bacteriology and Virology, School of Medicine, Shiraz University of Medical Sciences, Shiraz, Iran;; 3 Department of Virology, School of Medical Sciences, Tarbiat Modarres University, Tehran, Iran;; 4 Shiraz Institute for Cancer Research, School of Medicine, Shiraz University of Medical Sciences, Shiraz, Iran;; 5 Gastroenterohepatology Research Center, Shiraz University of Medical Sciences, Shiraz, Iran;; 6 Infectious Diseases Research Center, Golestan University of Medical Sciences, Gorgan, Iran

**Keywords:** Herpesvirus 4, Tumor suppressor protein p53, USP7 protein

## Abstract

Background:

The *p53* mutation is uncommon in EBV-linked gastric carcinoma, but its suppression occurs through mechanisms such as USP7 inhibition via EBNA1 activity. This study aimed to evaluate the effect of EBNA1 on *p53*-inhibiting gene expression and the impact of USP7 inhibition on *p53* suppression.

**Methods:**

MKN-45 cells were transfected with the *EBNA1 *plasmid. A stable EBNA1 expression cell line was developed through selection based on hygromycin B resistance. *MDM4*, *MDM2*, *SIRT3*, *HDAC1*, *PSMD10*, *USP7*, and *p53* expression were checked using real-time PCR. Also, cells containing EBNA1 or control plasmid were treated with GNE-6776, and the expression of the interested genes and cell survival were assessed.

**Results:**

* MDM4*, *MDM2*, and *PSMD10* were significantly upregulated in the MKN-45 cell line following *EBNA1* transfection. Morphological changes were observed in the cells harboring *EBNA1* after 20 days. In the control cells, USP7 inhibition significantly upregulated the *HDAC1*, *PSMD10*, *MDM4*, and *MDM2* genes after 24 h, but downregulated these genes after four days. In the *EBNA1*-harboring cells, *MDM2*, *MDM4*, and *PSMD10* genes were significantly upregulated after 24 h, and this effect was sustained for all genes except for *MDM4*, even after four days. Furthermore, USP7 inhibition induced apoptosis in both cell groups.

**Conclusion:**

EBNA1 enhances the expression of p53-inhibiting genes. Two events—p53 protein overexpression and apoptosis activation—followed the suppression of the USP7 protein and provided evidence for its possible function. The significance of the EBNA1-USP7 interaction in p53 suppression warrants additional investigation and possibly reconsideration.

## INTRODUCTION

Epstein–Barr virus, commonly referred to human herpesvirus 4, is regarded as an oncogenic double-stranded DNA virus (length ~170 kb), identified in 8-10% of gastric adenocarcinomas^[^^1^^]^. Although the infection rate of EBV in individuals over 30 years of age is 95%, the vast majority of adults merely carry the virus until the end of their lives, with no risk of malignancy^[^^2^^]^. In contrast to certain oncogenic viruses like the human papillomavirus, EBV genomic components typically do not integrate into the host genome^[^^3^^]^. EBV episomes have nucleosomal structures with capability of replication and viral gene expression^[^^4^^]^.

While* p53* mutations are uncommon in EBV-linked gastric adenocarcinoma tissues, they are frequently detected in other kinds of gastric carcinoma^[^^5^^]^. Several EBV-encoded products have been shown to inhibit the activities of p53 as a tumor suppressor during viral latency in EBV-associated cancers^[^^6^^]^. Various EBV-encoded products have been implicated in inactivating pathways mediated by p53^[^^7^^]^. EBNA1 is the only viral protein in all EBV latency types^[^^8^^]^. This protein, as a DNA-binding transcription factor, has regulatory roles in the transcription of viral and host promoters^[^^8^^]^. EBNA1 protein binds to numerous DNA sequences in the cellular genome, including the promoters of the genes whose transcription is regulated by EBNA1^[^^9^^, ^^10^^]^.

USP7 (known as HAUSP) is a deubiquitinating enzyme recently discovered as a critical regulator of the p53-MDM2 pathway, in which both *p53* and *MDM2* are stabilized by this enzyme^[^^11^^]^. It has been reported that the EBNA1 protein binds to USP7 with high affinity and impairs the interaction of p53 and USP7. The p53 continues to be ubiquitinated and destroyed by the proteasome after this interaction^[^^12^^]^. USP7 is also associated with the stability of negative p53 regulators, MDM4 and MDM2, showing its contradictory function in p53 control^[^^11^^,^^13^^]^. Inhibition of USP7 may kill cancer cells by restoring p53 and inducing apoptosis^[^^13^^]^. Besides, USP7 inhibitory activity reduces *MDM2* expression. Indeed, USP7 can affect gene expression by regulating transcription factors. 

The question of whether the EBNA1 protein is involved in viral-associated tumorigenesis has been extensively argued. However, growing evidence has pointed to the oncogenic activity of EBNA1 and the importance of this protein in inhibiting p53 activities and decreasing p53 activation^[^^7^^]^. The overexpression of some *p53*-inhibiting genes, such as *MDM4*,* MDM2*,* PSMD10* or* gankyrin*,* HDAC1*, and* SIRT3*, whose transcription may be regulated by EBNA1, play an important role in wild-type p53 suppression. MDM4 and MDM2 affect p53 by regulating its activity and stability^[^^14^^]^. PSMD10 decreases cell apoptosis by p53 degradation^[^^15^^]^. HDAC1 and SIRT3 can influence the function of p53 by its deacetylation^[^^16^^,^^17^^]^. This research analyzed the mRNA expression of p53-inhibiting genes and p53 mRNA/protein in a human gastric cancer cell line transfected with *EBNA1*, based on the idea that EBNA1 might affect the promoter of p53-inhibiting genes. We also examined the impact of USP7 inhibition on the expression of p53-inhibiting genes and then on p53 in the presence or absence of EBNA1 protein.

## MATERIALS AND METHODS

**Cell line, transfection, and selection of transfected cells**

MKN-45, an EBV-negative human gastric adenocarcinoma cell line with wild-type *p53 *and no pathological single-nucleotide polymorphisms in the* p53* gene, was purchased from the National Cell Bank of the Pasteur Institute of Iran, Tehran. MKN-45 cell line was routinely cultured in a medium containing 80% RPMI1640 and 20% FBS. A total of 6 × 10^4 ^cells were seeded in a six-well plate and grown for 24 hours. Plasmid PCEP4 (Invitrogen, USA) carrying the EBV replication origin (OriP) and *EBNA1* (strain B95.8) was used for stable transfection. As a control, a plasmid missing the EBNA1 gene was used. The transfection techniques were performed according to the manufacturer's guidelines using Lipofectamine 2000 (Invitrogen). After 24 hours of transfection, 350 µg/mL of hygromycin B, based on the Invitrogen manual (PCEP4, Catalog no. V044-50), was supplemented to the cell culture environment for 16 days to select the transfected cells with stable *EBNA1* expression.

**Total RNA extraction and cDNA synthesis**

Total RNA was extracted using an RNA isolation kit (Dena Zist, Iran). Spectrophotometry (Nanodrop^TM ^Spectrophotometer, Thermo Scientific, USA( and gel electrophoresis were used to assess the quantity and quality of the extracted RNA, respectively. Isolated RNA (1 µg/µLl from each sample) was converted into cDNA with an EasycDNA Synthesis kit (Parstos, Iran) following the manufacturer’s instructions.

**Primer design and real-time PCR**

Primers were designed based on the exon-exon junction and intron spanning methods using primer designing software and the NCBI gene database. The sequences of the primers are shown in Table 1. The *EBNA1* gene expression was confirmed using SYBR green-based real-time PCR, and the expression of the *HDAC-1*,* SIRT3*,* PSMD10*,* p53*,* MDM2, MDM4*, and *USP7* genes was quantified using the same method. The real-time PCR ABI 7500 apparatus (Applied Biosystems, Grand Island, New York, United States) was used for gene expression evaluation. The *beta-actin* gene was utilized as the reference gene^[^^18^^]^. Each reaction contained 2× Master Mix Green (Ampliqon Inc., Denmark), cDNA (each primer at a 10-pmoL concentration), and water in a final volume of 15 µL. PCR program began with a 15-minute denaturation phase at 95 °C, followed by 40 cycles of 95 °C for 15 seconds and annealing/extension at 62 °C (for the* SIRT3 *gene at 58 °C) for 1 minute. The melting curves of all the amplifications were analyzed and then it was validated that one target has been amplified in each gene test reaction, and no primer dimer was formed. By running real-time PCR products on gel electrophoresis, the size of the products was confirmed. Moreover, standard curves for all genes were drawn to verify that all primers bind to and amplify their targets efficiently.

**Verification of** ***EBNA1*** **gene expression**

According to the manufacturer’s procedure for removing the plasmid contamination, the total RNA isolated from the transfected cells was treated with RNase-free DNase (Sinaclon, Tehran, Iran). EBNA1 expression was confirmed using real-time PCR. DNase-treated whole RNA was utilized as the negative control.

**Determination of cell toxicity of GNE-6776**

Two different techniques, PI spectrofluorometry and trypan blue exclusion assays, were utilized to evaluate cell viability to demonstrate that 15 µM of GNE-6776 (*USP7*inhibitor; MedChemExpress, USA) is non-toxic, for the MKN-45 cells. In the trypan blue exclusion assay, MKN-45 cells were seeded in a 24-well plate (1 × 10^5^ cells per well) and incubated for 24 hours. The cells were then treated with different concentrations of GNE-6776 (15, 30, 50, 70, and 100 µM) for 24 hours. Negative control cells were left untreated, while positive control cells were generated by treating MKN-45 with a toxic concentration of dimethyl sulfoxide. Trypan blue (0.4%) and MKN-45 cells (1:1) were combined, and the vitality of the cells was determined using a hemocytometer slide; the assay was performed in triplicate. In the second trial, MKN-45 cells were seeded in a 96-well plate (1 × 10^4^ cells per well) and incubated for 24 hours. MKN-45 cells were incubated for further 24 hours following treatment with 30, 50, 70, or 100 µM of GNE-6776. The positive control cells were treated with dimethyl sulfoxide, and the negative control ones were left untreated. The test was conducted in triplicates. Media lacking cells were utilized as blank. Each well received 5 µg of PI reagent and was incubated at room temperature for 10 minutes. 

**Table 1:** Primers used for evaluation of the gene expression by relative quantitative real-time PCR

**Gene **	**Sequences**	**Product size (bp)**
*MDM2*	5΄-AACCACCTCACAGATTCCA-3΄ 5΄-GCACCAACAGACTTTAATAACTTC-3΄	87
		
*PSMD10*	5΄-CTACTAGAACTGACCAGGACA-3΄ 5΄-GCCGCAATATGAAGAGGAG-3΄	145
		
*SIRT3*	5΄-ACTCCCATTCTTCTTTCACAAC-3΄ 5΄-GGATGCCCGACACTCT-3΄	176
		
*USP7*	5΄-TGGTGGAGCGATTACAAGA-3΄ 5΄-TCCTCTGCGACTATCTGC-3΄	100
		
*Beta-actin* ^[^^18^^]^	5΄-GCCTTTGCCGATCCGC-3΄ 5΄-GCCGTAGCCGTTGTCG-3΄	90
		
*TP53*	5΄-GATAGCGATGGTCTGGC-3΄ 5΄-CGGCTCATAGGGCACC-3΄	117
		
*HDAC1*	5΄-GACGGTAGGGACGGGAG-3΄ 5΄-GGCTTTGTGAGGGCGATAG-3΄	203
		
*EBNA1*	5΄-GGGTGGTTTGGAAAGCATCG-3΄ 5΄-CTTACTACCTCCATATACGAACACA-3΄	156
		
*MDM4*	5΄-GCCTGCCTTGGTGGTT-3΄ 5΄-CCTAACTGCTCTGATACTGACTC-3΄	160

Fluorescence intensity was measured with a detection microplate reader (FLUOstar Omega, BMG LABTECH, Germany) for PI at the excitation and emission wavelengths of 544 nm and 622 nm, respectively; the assay was performed in triplicate.

**USP7 inhibitor treatment**

The total number of 5 × 10^5^ cells, including the untransfected, control plasmid transfected, and PCEP4-transfected cells (selected using hygromycin B within 20 days), were seeded into a six-well plate separately. GNE-6776 (15 µM; MedchemExpress) was used as a suppressor rephrase. Cells were collected at two different time points (days 1 and 4), and the total RNA was isolated to evaluate the effect of USP7 inhibition on *p53* and *p53*-inhibiting gene expression.

**Pathology staining and immunocytochemistry**

After 20 days of transfection and selection with hygromycin B, the transfected cells were collected, and EBNA1 expression was validated at the mRNA level by real-time PCR in the *EBNA1* plasmid-containing cells. The morphological alterations were then examined using pathological staining. For the *p53* expression test, the semi-adherent cells were collected, centrifuged, and fixed in formalin. Slices of paraffin-embedded cells were cut and then stained using a pre-diluted primary anti-p53 antibody before evaluation by optical microscopy.

**Cell viability analysis of** ***EBNA1*** **harboring and negative cells**

In a 12-well plate, MKN-45 cells, including untransfected, control plasmid transfected (no EBNA1 expression), and PCEP4 transfected (stable EBNA1 expression), were seeded (2 × 10^5^ cells per well). After 24 hours, each well was treated with 15 µM of GNE-6776 antagonist and then incubated for another 24 hours. The cells in each well were then treated with AO/PI reagent prior to a 10-minute incubation at room temperature. A fluorescent microscope was used to assess the outcomes (FLUOstar Omega).

**Data analysis**

First, the Ct values of real-time PCR runs were equalized using the CtNormalgorithm (http://ctnorm. sums.ac.ir)^[19]^. After Ct normalization, the data were computed in Microsoft Excel (Microsoft Office Professional Plus 2016, Microsoft Corporation, Washington, USA). Mann–Whitney U test was used to compare the means in GraphPad Prism version 5.0. This software was used to calculate the 50% cell viability following GNE-6776 treatment. *P *values below 0.05 were considered statistically significant.

## RESULTS

**Pathological staining for morphologic examination**

**Fig.1:**
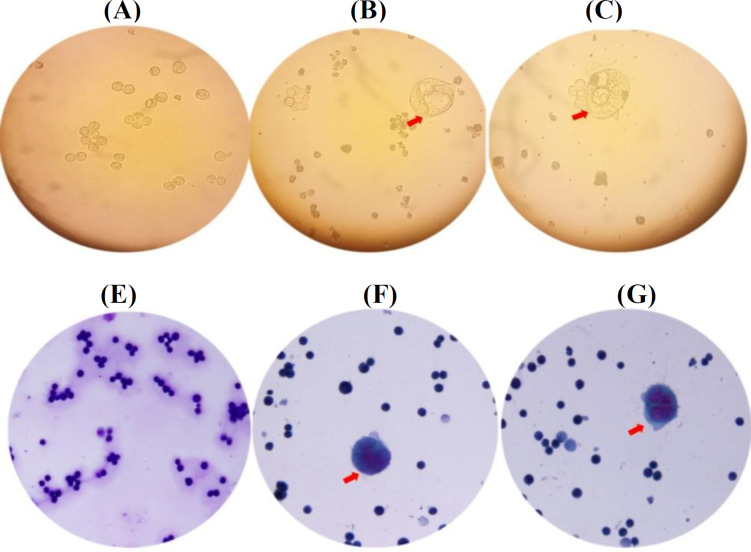
Morphological changes of MKN-45 cell lines transfected with *EBNA1* and control plasmid. (A) Cell line transfected with control plasmid after 20 days; (B and C) abnormal morphology (abnormal cells were indicated by arrows) in the culture of the MKN-45 cell line transfected with *EBNA1* after 20 days; (D) pathological staining of control plasmid-transfected cell after 20 days; (E and F) pathological staining of *EBNA1* plasmid-harboring cell after 20 days

Following the selection of hygromycin B-resistant clones, we noticed morphological alterations in *EBNA1*-transfected cells. Comparing the *EBNA1* transfected cells to the control cells revealed some morphological differences using an inverted microscope (Fig. 1A-1C). Pathological staining showed that *EBNA1 *expression altered the MKN-45 cell shape compared with the control (plasmid-transfected) cells (Fig. 1D-1F). 

**Expression of** ***p53*** **-inhibiting genes following** ***EBNA1*** **transfection**

**Fig. 2:**
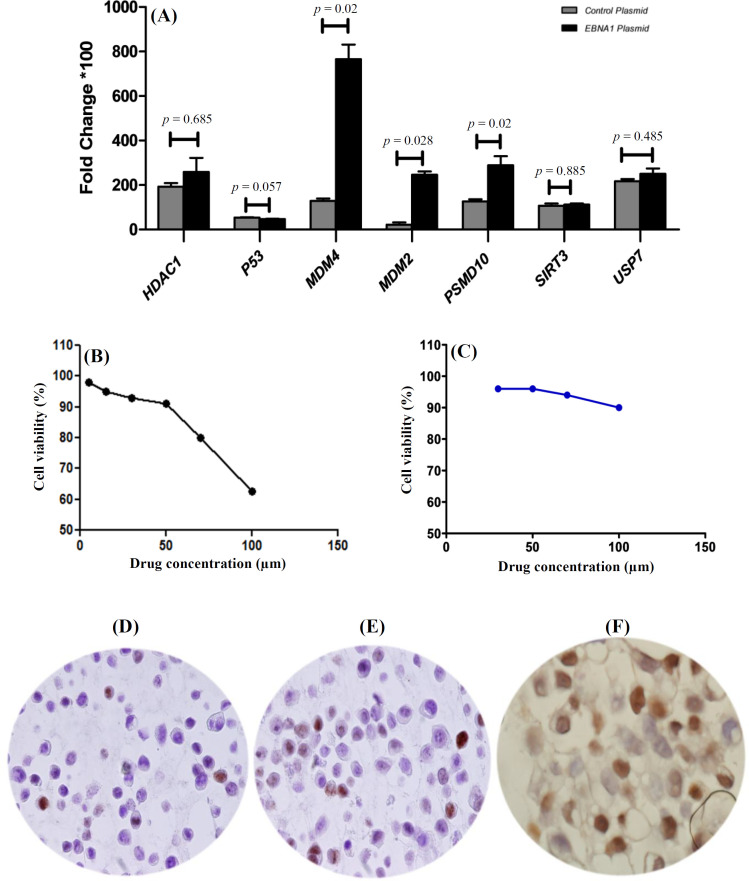
Expression of *p53*-inhibiting genes in MKN-45 cells, and immunocytochemistry (ICC) for p53 and PI/Trypan blue exclusion assays. (A) *p53*-inhibiting genes expression in MKN-45 cell line after transfection with *EBNA1* plasmid; (B) Trypan blue exclusion assay and (C) PI spectrofluorometry for cell viability assay, showing that 15 µM of GNE-6776 is nontoxic for the MKN-45 cells. ICC for p53 in MKN-45 cells transfected with (D) the control plasmid and (E) *EBNA1 *plasmid; (F) ICC for p53 in MKN-45 cells transfected with *EBNA1* plasmid and treated with GNE-6776, p53 upregulated in MKN-45 cell-harboring *EBNA1* plasmid following GNE6776 treatment

The expression levels of the seven genes of interest (*MDM2*, *MDM4*, *PSMD10*, *SIRT3*, *HDAC1*, *USP7*, and *p53*) were compared between *EBNA1*-transfected and plasmid-transfected cells (Fig. 2A). The mRNA expression levels of *MDM4* (*p *= 0.02), *MDM2* (*p *= 0.028), and *PSMD10 *(*p *= 0.02) were significantly upregulated in MKN-45 cells transfected with *EBNA1* compared to the plasmid-transfected controls. However, the analysis showed no significant changes in the mRNA expression of *HDAC1 *(*p *= 0.685), *SIRT3 *(*p *= 0.885), and *USP7 *(*p *= 0.485) in the *EBNA1*-transfected cells compared to the control cells. Furthermore, *p53* mRNA expression decreased insignificantly in the MKN-45 cells transfected with *EBNA1* (*p *= 0.057; Fig. 2A).

**Effects of** ***USP7*** **inhibitor on mRNA expression of** ***p53*** **-inhibiting genes in** ***EBNA1*** **-transfected cells**

**Fig. 3:**
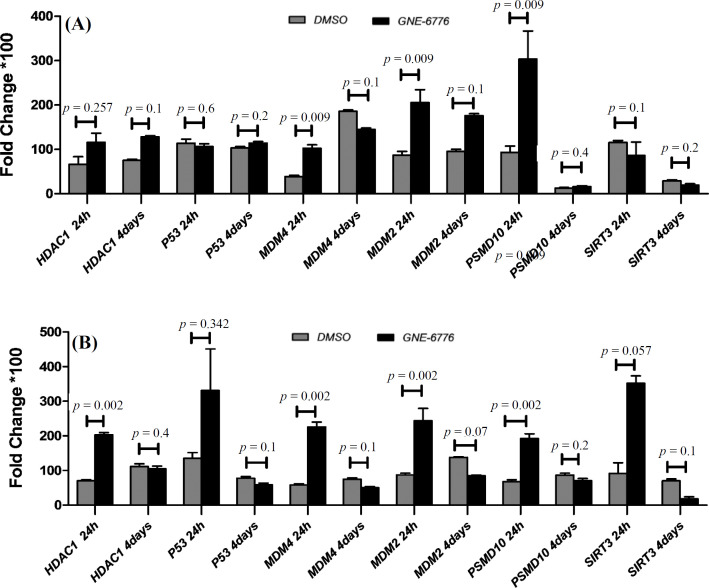
Evaluation of *p53*-inhibiting genes expression following treatment of *EBNA1*-positive and -negative cells with GNE-6776 after 24 hours and after four days by quantitative PCR. (A) The cells harboring *EBNAl* plasmid were treated with GNE6776 (test) and DMSO (control) to evaluate the effect of USP7 inhibition on p53 and *p53*-inhibiting genes in the presence of *EBNA1*; (B) effects of USP7 inhibitor on mRNA expression of *p53*-inhibiting genes in the control plasmid-transfected cell. The cells harboring control plasmid were treated with GNE6776 (test) and DMSO (control) to evaluate the general effect of USP7 inhibition on p53 and *p53*-inhibiting genes

Both of the toxicity assays showed that 15 µM of GNE-6776 was nontoxic for MKN-45 cells (Figs. 2B and 2C). Thus, *EBNA1*-harboring cells were treated with GNE-6776 at 15-µM final concentration. After 24 hours, the real-time PCR assay demonstrated that the expression levels of all genes except for *SIRT3* (*p *= 0.1) were greater in the USP7 inhibitor-treated MKN-45 *EBNA1*-transfected cells than those in the untreated cells. A significant upregulation was observed in the expression of *MDM4* (*p *= 0.009), *MDM2 *(*p *= 0.009), and *PSMD10 *(*p *= 0.009), but not that of *HDAC1 *(*p *= 0.257). Furthermore, the expression of *p53 *(*p *= 0.6) was insignificant in the treated cells (Fig. 3A). After four days of treatment of *EBNA1*-transfected cells with GNE-6776, the expression levels of *PSMD10 *(*p *= 0.4), *HDAC-1 *(*p *= 0.1), and *MDM2 *(*p *= 0.1) genes were not significantly higher than the untreated cells. Although the expression of *MDM4* (*p *= 0.009) was upregulated after 24 h, it was downregulated after four days (*p *= 0.1). The mRNA expression of *p53* was also upregulated after four days (*p *= 0.2; Fig. 3B).

**Effects of** ***USP7*** **inhibitor on mRNA expression of** ***p53*** **-inhibiting genes in the control plasmid-transfected cell**

To examine the effect of inhibiting the ubiquitination activity of *USP7* on *p53* and its inhibitor gene expression, the control plasmid-transfected cells were treated with the *USP7 *inhibitor. Figure 3B indicates the upregulated expression of *HDAC1 *(*p *= 0.002), *PSMD10 *(*p *= 0.002), *SIRT3 *(*p *= 0.057), *MDM4* (*p *= 0.002), and *MDM2 *(*p *= 0.002) after 24 h, but they were downregulated after four days. Thus, mRNA expression of *p53* was upregulated and downregulated after 24 h (*p *= 0.342) and four days (*p *= 0.1), respectively (Fig. 3A).

**Effects of** ***USP7*** **inhibitor on mRNA expression of** ***p53*** **inhibitor genes in the** ***EBNA1*** **-transfected vs. the control plasmid-transfected cells**

**Fig. 4:**
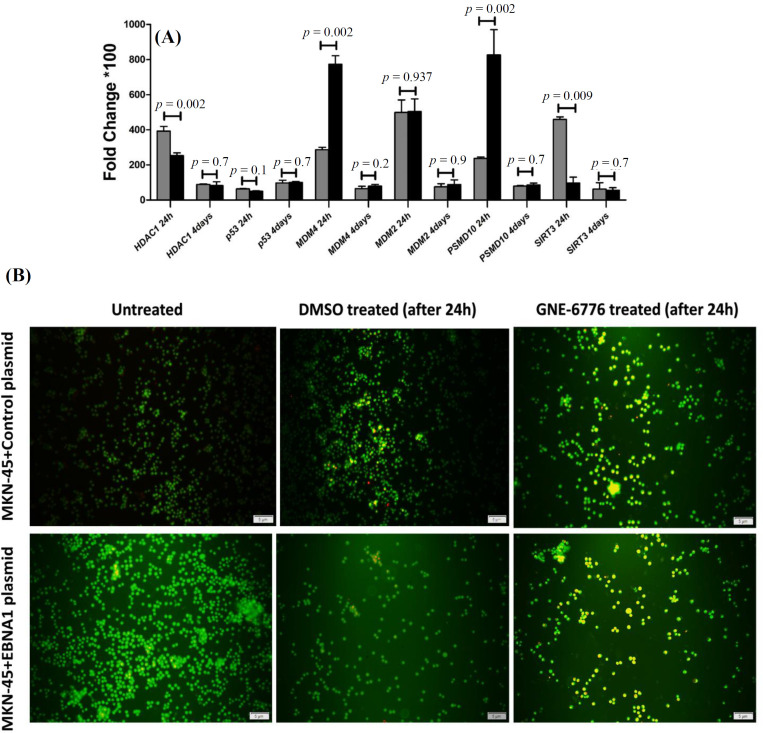
Comparison effect of GNE6776 on the cell harboring *EBNA1* plasmid vs. the cell harboring control plasmid and evaluation of GNE-6776 treatment on cell viability by AO/PI. (A) Effects of USP7 inhibitor on the cell harboring *EBNA1* plasmid and control plasmid harboring cell after 24 hours and 4 days; (B) AO/PI test for cell viability after GNE-6776 treatment. The cell harboring control plasmid group had higher yellow cells (in early apoptosis) compared to the cell harboring *EBNA1* plasmid group following treatment with GNE-6776

Comparing the mRNA expression of *p53*-inhibiting genes between *EBNA1*-transfected cells and control plasmid-transfected cells revealed that *PSMD10 *(*p *= 0.002) and *MDM4 *(*p *= 0.002) were significantly expressed in the *EBNA1*-harboring cells after 24 h. Expression of *HDAC1* (*p *= 0.002) and *SIRT3* (*p* = 0.009) was significantly higher in control plasmid-harboring cells after 24 h than *EBNA1*-containing cells. Nevertheless, after four days, there was no discernible difference between EBNA1-containing cells and controls at the mRNA expression level of the selected genes. Although *p53* mRNA expression was lower in* EBNA1* transfected cells than in the control cells, it was nonsignificant (*p *= 0.1). Therefore, after four days, *p53* mRNA expression was not significantly different between two groups (Fig. 4A).

**Immunocytochemistry test for** ***p53*** **expression**

Immunocytochemical analysis with an optical microscope showed positive expression of the p53 protein in the MKN-45 cells transfected with the control plasmid (15%-20%), MKN-45 cells harboring *EBNA1 *(25%), and MKN-45 cells harboring *EBNA1 *+ GNE6776 treatment (50%-60%), as depicted in Figures 2D-2F.

**Cell viability analysis of** ***EBNA1*** **-transfected cells and control plasmid-transfected cells**

In AO/PI staining, green, yellow, and red fluorescence indicate live cells, early apoptosis, and late apoptosis (necrosis), respectively. After 24 hours, fluorescence microscopy examination revealed an increase in yellow cells (early apoptosis) in the GNE-6776-treated groups. Additionally, the AO/PI assay demonstrated that GNE-6776 caused apoptosis in both *EBNA1* plasmid- and control plasmid-transfected cells. However, as shown in Figure 4B, the *EBNA1*-transfected cells exhibited a somewhat slower progression of apoptosis (fewer yellow cells).

## DISCUSSION

Our study indicates that all surveyed*p53*-inhibiting genes, including *MDM2*, *MDM4*, *HDAC1*, *SIRT3,* and *PSMD10,* were upregulate at the mRNA level in MKN-45 *EBNA1*-transfected cells compared with MKN-45 plasmid-transfected control cells. However, this upregulation is statistically significant in only three of these genes, namely *MDM4*,* MDM2*, and* PSMD10*. Moreover, *p53* mRNA expression decreased after *EBNA1* transfection. These results demonstrate an association between EBNA1 expression and the expression of p53-inhibiting genes in the MKN-45 cell line, suggesting p53 suppression following upregulation of negative regulators.

According to our findings, the mRNA level of p53 was downregulated in the cells transfected with *EBNA1*. The immunocytochemistry tests revealed that the p53 protein level is about 5% higher in these cells than in the control cells. Similarly, Ribeiro et al.^[^^20^^]^ found that EBV-associated gastric carcinomas substantially reduced the *p53* mRNA levels with high p53 protein in the IHC tests, and *p53* mutations were infrequent in EBV-positive gastric carcinomas. One interpretation of this event is that the function of p53 protein is likely inhibited by the upregulation of some p53-inhibiting genes, leading to its accumulation in the cell. Our results and Ribeiro et al.’s^[^^18^^]^ study showed that EBV modulates p53 mRNA expression and its protein accumulation, but more research is required to pinpoint the precise mechanisms involved. 

Pathological staining reveals that *EBNA1* expression influences the MKN-45 cell shape. In the same line, Wang et al.^[^^21^^]^ explored that the EBNA1 protein was significantly expressed in NPC tissue samples, linking this expression with NPC lymph node metastasis^[^^21^^]^. They found that morphology of NPC cells and the expression of markers for the epithelial-mesenchymal transition were both impacted by EBNA1 expression *in vitro*. As a result, the findings of our study and that of Wang et al.^[^^21^^]^ showed that the EBNA1 protein by itself can help cancer progression. We suggest that the morphological changes induced by *EBNA1* in NPC and adenocarcinoma cell lines need more consideration and evaluation.

The results of the current study showed that the transcript levels of the histone deacetylase genes *HDAC1* and *SIRT3* have also increased, but these changes are not statistically significant. A study found that DNMT3a/b/L and a group of HDACs had higher expression levels in the EBV-positive cells^[^^22^^]^. Similarly, Edwards et al.^[^^23^^]^ have found that HDAC1 is activated in the EBV-positive tumors in AGS-EBV malignancies, highlighting the importance of epigenetic regulation during tumor growth. A related mechanism is that HDAC1 increases p53 degradation by eliminating the acetyl groups^[^^24^^]^. We showed that the *SIRT3* gene is insignificantly upregulated in the *EBNA1* transfected cells. The function of this gene may vary depending on the cell and tumor type; as a result, *SIRT3* may operate as an oncogene or a tumor suppressor^[^^25^^]^. Some viruses have evolved p53 deacetylation mechanisms, including the upregulation of *SIRT*s, which makes p53 inactive, enabling the cell to survive and the virus to spread^[^^26^^]^. Consequently, blocking SIRTs to restore wild-type p53 transcriptional activity in tumors that still express normal p53, may be a potential therapeutic strategy, especially when combine with conventional treatments^[^^27^^]^.

The* MDM2* and *MDM4* genes are negative p53 tumor suppressor protein regulators. The MDM4 protein interacts with p53 and inhibits its action. Both MDM2 and MDM4 proteins are overexpressed in a range of human cancers. Our research demonstrated that *EBNA1*-transfected cells expressed significantly higher *MDM2* and *MDM4* mRNA levels than the control cell lines (especially *MDM4*). It has also been shown that EBV is linked with increased *MDM2* expression^[^^28^^]^. Renouf et al.^[^^29^^]^ surveyed the effects of nutlin-3, a specific inhibitor of the p53-MDM2 interaction that stabilizes and activates p53, in combination with different chemotherapeutic drugs. They have reported that nutlin-3 sensitizes EBV-negative and latency I EBV-positive Burkitt’s lymphoma cells to these drugs. They have indicated that activating *p53* with *MDM2 *antagonists has distinct apoptotic effects on EBV-positive and EBV-negative Burkitt’s lymphoma cell lines^[^^29^^]^. AlQarni et al.^[^^28^^] ^have implied that *EµEBNA1 *tumor cells rely on not only c-MYC but also MDM2 for survival, and that *MDM2* suppression does not result in *p53* overexpression; instead, a decline in E2F1 expression is linked to cell death. They have also demonstrated that several *MDM2* isoforms are elevated in the *EµEBNA*1 tumors^[^^28^^]^. The CRISPR screen assay shows that *MDM2* and *MDM4* as *p53* inhibitors are required for the survival of established lymphoblastoid cell lines^[^^30^^]^. The p53-MDM4 pathway is crucial for reacting to DNA damage and preventing the development of cancer^[^^31^^]^. *MDM4* overexpression has been associated with tumor formation and a poorer prognosis^[^^32^^]^. Based on these findings, we predict that EBNA1 may be linked to *MDM2* and *MDM4* expression levels, as well as the risk of developing gastric adenocarcinoma.

The* PSMD10 *gene, which encodes the gankyrin protein, is a regulatory component of the 26S proteasome. Ubiquitin-dependent protein degradation necessitates the presence of 26S proteasome complex. The erroneous expression of this gene may play a part in the tumorigenesis^[^^33^^]^. As a proto-oncoprotein, it negatively regulates the tumor suppressors RB1 and p53^[^^33^^]^. According to our findings,* PSMD10* is substantially overexpressed after *EBNA1* transfection. Kashyap et al.^[^^34^^] ^exhibited that *Helicobacter pylori* and EBV co-infection increased the aggressiveness of gastric cancer via *gankyrin *upregulation. Enhanced *gankyrin* expression was related to disease development and metastasis in a variety of malignancies, making it a potential target for cancer treatment^[^^35^^]^. Therefore, the upregulation of *PSMD10* gene reported in EBV-associated gastric cancer maybe associated with the expression of *EBNA1* in cancerous cells infected with EBV.

USP7 enzyme deubiquitinates its target proteins. USP7 has a perplexing role in regulating p53 activities in several ways. USP7 binds to and directly deubiquitinates p53 and inhibits its degradation. On the other hand, USP7 interacts with MDM2 to improve its stability by deleting ubiquitin on MDM2 and protecting it from proteasome destruction^[^^36^^]^. MDM2 has a higher affinity for USP7^[^^37^^]^. Although EBNA1 binds to USP7, neither EBNA1 turnover nor cell-surface expression is influenced by this interaction^[^^38^^]^. It has been observed that p53 and EBNA1 have similar binding sites on USP7 and effectively compete for USP7 binding, resulting in decreased p53 stability and protection against apoptosis^[^^38^^]^. In contrast, according to certain research, specific USP7 inhibition causes cancer cell death via p53-dependent mechanism^[^^39^^]^. Our data indicated that USP7 increases in cells transfected with *EBNA1*. Wang et al.^[^^39^^] ^discovered that USP7 directly interacted with PD-L1 and maintained it. Also, deleting USP7 made cancer cells more vulnerable to T cell death, both *in vitro* and *in vivo*. In addition, USP7 inhibitors suppressed the development of gastric cancer cells *in vitro* and *in vivo* via stabilizing p53^[^^39^^]^.

In the second phase of the research, we used GNE-6776 to survey the effects of USP7 inhibition on the *EBNA1 *plasmid and control plasmid-transfected cells. All *p53*-inhibiting genes (*HDAC1*, *SIRT3*, *PSMD10*, *MDM2*, and *MDM4*) examined in our study were upregulated after 24 hours in the *EBNA1*-negative MKN-45 cell line (four genes including *HDAC1*,* MDM2*,* MDM4*, and *PSMD10* to a significant degree), but they were not upregulated significantly after four days. Following 24 hours of treatment with GNE-6776 in the* EBNA1*-transfected MKN-45 cell line, the *MDM2*, *HDAC1*, and *PSMD10 *expression levels increased, but after four days, their expression did not raise significantly. Furthermore, after being treated with GNE-6776, the *EBNA1*-plasmid transfected cells had higher mRNA levels of *MDM2* and *PSMD10* than the control plasmid cells. In the cells harboring the control plasmid,* HDAC1* and *SIRT3* had greater mRNA levels than *EBNA1 *plasmid-harboring cells after GNE-6776 treatment. After four days, there was no significant change between two cell groups. The AO/PI assay demonstrated that GNE-6776 promotes apoptosis in both *EBNA1* and control plasmid-transfected cells. However, the *EBNA1*-transfected cells exhibited lower apoptosis progression based on the percentage of yellow cells in each field under the fluorescence microscope*.* Immunocytochemistry of *p53* in the *EBNA1*-transfected cells treated with GNE-6776 revealed that after suppressing USP7, p53 levels increased by 50-60%. Thus, based on our results, suppression of *USP7* by GNE6776 is associated with increases in *p53* expression at the protein level and apoptosis induction in the *EBNA1 *plasmid/control plasmid-transfected cells carrying the *p53* wild type. Consequently, after four days of USP7 inhibition, we found no statistically significant increase in the expression of *p53*-inhibiting genes at the mRNA level; however, we did find apoptosis and a rise in or stability of p53 at the protein level. Although we identified several differentially expressed mRNAs between transfected and un-transfected cell lines, some limitations are present. In follow-up research, the identified proteins should be validated using additional methods like Western blotting. Moreover, the unavailability of other gastric adenocarcinoma cell lines for further investigation and comparison with the results in the MKN-45 cell line is another limitation of the study.

In conclusion, our research indicates that the EBNA1 protein is likely related to the upregulation of some* p53*-inhibiting genes in the *EBNA1*-harboring cells at the mRNA level, and upregulation of *p53*-inhibiting gene collection may be a key mechanism in EBV-associated gastric adenocarcinoma (latency type I). Therefore, we recommend performing more investigations on other cell lines or clinical samples (EBV-associated malignancy) to examine other p53-inhibiting/inducing genes at the mRNA and protein levels, which will provide unique insights into the biological activities of EBNA1 in carcinogenesis. Furthermore, our results imply that additional research is necessary to fully understand the role that EBNA1-USP7 interaction plays in the p53 suppression in gastric cancer, and that it should also be given another look.

## DECLARATIONS

### Acknowledgments

The present study was extracted from a Ph.D. thesis written by Seyed Mohammad Ali Hashemi. 

### Ethical statement

Not applicable.

### Data availability

The analyzed data sets generated during the study are available from the corresponding author on reasonable request.

### Author contributions

SMAH: study concept, bench work, data analysis, and manuscript drafting; AM: study concept, scientific advice, and critical revision of the manuscript; SYH: study concept and scientific advice, and critical revision of the manuscript; SMHRN: study concept and critical revision of the manuscript; TB: scientific advice; ZF: scientific advice and critical revision of the manuscript; SJS: study concept and critical revision of the manuscript; AT: study concept, scientific advice, and critical revision of the manuscript. All authors contributed to the revision of the manuscript and approved the final manuscript.

### Conflict of interest

None declared.

### Funding/support

The present study was financially supported by Golestan University of Medical Sciences, Gorgan, Iran (no.111611; IR.GOUMS.REC.1400.130).
